# Increased Oxidative Stress as a Risk Factor in Chronic Idiopathic Axonal Polyneuropathy

**DOI:** 10.1007/s12031-018-1200-5

**Published:** 2018-10-22

**Authors:** Panagiotis Zis, Patrick C. McHugh, Maurizio Manca, Ptolemaios Georgios Sarrigiannis, Dasappaiah Ganesh Rao, Marios Hadjivassiliou

**Affiliations:** 1 Academic Department of Neurosciences, Royal Hallamshire Hospital, Glossop Rd, Sheffield, South Yorkshire S10 2JF UK; 2 Centre for Biomarker Research and Department of Pharmacy, School of Applied Sciences, University of Huddersfield, Queensgate, Huddersfield, HD1 3DH UK

**Keywords:** Chronic idiopathic axonal polyneuropathy, CIAP, Gluten neuropathy, Pathogenesis, Oxidative stress

## Abstract

Chronic idiopathic axonal polyneuropathy (CIAP) is a disorder with insidious onset and slow progression, where no etiology is identified despite appropriate investigations. We aimed to investigate the role of oxidative stress as a risk factor for the pathogenesis of CIAP. Sera of patients with CIAP were tested for protein carbonyl (PC) and 8-hydroxydeoxyguanosine (8H). As a control group, we recruited patients with gluten neuropathy. Twenty-one patients with CIAP and 21 controls were recruited. The two groups did not differ significantly regarding demographics or clinical characteristics (i.e., neuropathy type or disease severity). After adjusting for gender, having CIAP was positively correlated with both the 8H titer (standardized beta coefficient 0.349, *p* = 0.013) and the PC titer (standardized beta coefficient 0.469, *p* = 0.001). Oxidative stress appears to be increased in CIAP and might have a role in the pathogenesis of the disease.

## Introduction

Chronic idiopathic axonal polyneuropathy (CIAP) is a term describing neuropathies where neurophysiology reveals axonal damage, their onset is insidious and shows slow or no progression of the disease over at least 6 months and no etiology is identified despite appropriate investigations (Zis et al. 2016). The majority of CIAP patients have a sensorimotor axonal peripheral neuropathy. The second commonest idiopathic large fiber polyneuropathy where axons degenerate is the sensory ganglionopathy (SG) (Zis et al. 2016).

Robust epidemiological data on polyneuropathies of any cause are lacking. Very few studies have accurately assessed the prevalence of polyneuropathy in the general population. The current estimates are between 5.5% (Hanewinckel et al. 2016) and 8.0% (Italian General Practitioner Study Group (IGPSG) 1995) in people more than 55 years. CIAP is reported to account for up to a third of polyneuropathies of any cause (Smith and Singleton 2004); however, the percentage of idiopathic polyneuropathies has declined with time (Singer et al. 2012) and the explanation for this is threefold. Firstly, with time and adequate follow-up, more causes of polyneuropathy—hereditary or acquired—have been recognized; secondly, more diagnostic tests have become available; thirdly, investigations about possible causes of polyneuropathy may have been incomplete (Zis et al. 2016).

Oxidative stress is increased in neurodegenerative diseases such as Alzheimer’s disease (Zis et al. 2012, 2014, 2017a; Zis and Strydom 2018) and it has been suggested that it plays a role in the pathogenesis of polyneuropathy in diabetes (Sifuentes-Franco et al. 2017), by causing damage to the lipids present in the myelinated structures of the nerves, resulting in the loss of axons and interruption of the microvasculature in the peripheral nervous system (Kasznicki et al. 2012).

The aim of this case-controlled study was to investigate the role of oxidative stress as a risk factor for the pathogenesis of CIAP.

## Methods

### Procedure and Participants

This was a cross-sectional case-controlled study.

Patients with CIAP were recruited from a dedicated neuromuscular clinic based at the Royal Hallamshire Hospital (Sheffield, UK). To be enrolled, the patients had to meet the following inclusion criteria: (1) clinical diagnosis of peripheral neuropathy (PN), confirmed on nerve conduction studies (NCS), (2) absence of other risk factors for developing PN (i.e., diabetes, vitamin deficiencies, exposure to neurotoxic agents), (3) normal results on an extensive diagnostic work-up (Zis et al. 2016), and (4) able to provide a written informed consent.

Individuals suffering from gluten neuropathy (GN) participated in the study forming a control group of patients with PN of a known etiology. To be enrolled, the controls had to meet the following inclusion criteria: (1) diagnosis of PN, as confirmed on nerve conduction studies, (2) serological evidence of gluten sensitivity (positive for antigliadin IgG and/or IgA with or without positivity for endomysial and transgultaminase antibodies) at diagnosis prior to commencing gluten-free diet, (3) absence of other risk factors for developing PN (i.e., diabetes, vitamin deficiencies, exposure to neurotoxic agents), and (4) able to provide a written informed consent.

The study protocol was approved by the local ethics committee.

### Measures

Demographic characteristics included age and gender. All patients and controls went through extensive investigations for possible causes of PN (Zis et al. 2016). Patients with a family history of neuropathy were excluded.

The type of neuropathy (sensorimotor length-dependent PN, sensory ganglionopathy (Zis et al. 2017b), mononeuritis multiplex) for all patients was determined based on nerve conduction studies, which were performed by the same clinician on the day of the recruitment.

The severity of neuropathy was assessed by the overall limitations neuropathy scale (ONLS), which is a validated scale that measures limitations in the everyday activities of the upper and lower limbs (Graham and Hughes 2006).

Oxidative stress markers in patients’ sera were analyzed through ELISA assay kits (Bioassay Technology Laboratory) according to manufacturers’ instruction. For the PC ELISA kit, the sensitivity was 1.07 ng/ml while for the 8H ELISA kit was 0.25 ng/ml.

In brief, 40 μl of sample was added to each well and mixed with 10 μl of antibody. After addition of 50 μl of streptavidin-HPR, the samples were incubated for 60 min at 37 °C followed by five washes with supplied buffer. After addition of 50 μl of substrate solution A and 50 μl of substrate solution B, the samples were further incubated at 37 °C for 10 min in the dark. Finally after the addition of 50 μl of stop solution, the samples’ optical density was assessed with an Infinite F50 (Tecan) plate reader at 450 nm. Each reading was plotted against the standard curve generated in the assay to retrieve the sample concentration.

### Statistical Analyses

A database was developed using the Statistical Package for Social Science (version 23.0 for Mac; SPSS). Frequencies and descriptive statistics were examined for each variable. Comparisons between CIAP and GN patients were made using Mann-Whitney’s *U* test for non-normally distributed and chi square or Fischer’s exact test for categorical data. Correlations between continuous variables were examined using Spearman’s tests. Multivariate regression analyses were performed when statistically significant differences or trends for statistically significant differences between groups were found in multiple variables.

The level of statistical significance was set at the 0.05 level.

## Results

Our study population consisted of 21 patients with CIAP and 21 patients with GN. The two groups did not differ significantly regarding age, gender, neuropathy type, and disease severity.

Neither PC nor 8H correlated significantly with age, neuropathy type, or disease severity in either group. Male patients, however, showed increased PC (168.6 ± 66.4 ng/ml versus 113.0 ± 21.3 ng/ml, *p* = 0.005) and increased 8H (29.2 ± 10.1 ng/ml versus 19.6 ± 3.3 ng/ml, *p* = 0.002) compared to females.

The univariate analysis showed that patients with CIAP had a significantly higher PC titer compared to GN patients (184.7 ± 73.9 ng/ml versus 126.0 ± 30.8 ng/ml, *p* = 0.012) indicating increased oxidative stress (Table 1).

**Table 1:** Demographic and clinical characteristics of our study population

	CIAP (*n* = 21)	GN (*n* = 21)	*p* value
Demographics			
Age, in years (SD)	71.4 (7.0)	68.2 (8.6)	0.290
Male gender (%)	16 (76.2)	16 (76.2)	1.000
Clinical characteristics			
Type of neuropathy			0.697
Sensorimotor axonal PN (%)	18 (85.7)	16 (76.2)	
Sensory ganglionopathy (%)	3 (14.3)	5 (23.8)	
Neuropathy severity			
ONLS arm score (SD)	1.1 (1.0)	1.1 (0.7)	0.989
ONLS Leg score (SD)	2.0 (1.0)	1.7 (1.3)	0.443
Total ONLS score (SD)	3.1 (1.8)	2.8 (1.6)	0.530
8-Hydroxy-desoxyguanosine (8H) titer (ng/ml)	30.4 (12.1)	23.5(5.3)	0.170
Protein carbonyl (PC) titer (ng/ml)	184.7 (73.9)	126.0 (30.8)	0.012

*CIAP*, chronic idiopathic axonal polyneuropathy; *GN*, gluten neuropathy; *SD*, standard deviation; *ONLS*, overall neuropathy limitations scale

After adjusting for gender, having CIAP was positively correlated with both the 8H titer (standardized beta coefficient 0.349, *p* = 0.013) and the PC titer (standardized beta coefficient 0.469, *p* = 0.001).

## Discussion

This case-controlled study demonstrates that patients with neuropathy of unknown etiology (CIAP) have significantly increased oxidative stress compared to a well-defined control group of patients with neuropathy of a known etiology (gluten neuropathy). This suggests that increased oxidative stress might be a risk factor for developing CIAP.

A strength of our study design is that we compared two groups of patients that did not differ regarding demographic and clinical characteristics. Moreover, our patients are under regular follow-up in our department since their diagnosis. By repeatedly performing extensive tests, as has been suggested for an accurate diagnosis of neuropathy of unknown etiology (Zis et al. 2016), we were able to accurately exclude patients with other comorbidities that increase the risk for the development of peripheral neuropathy (i.e., diabetes, excessive alcohol drinking, and B12 deficiency).

The role of oxidative stress has been studied in diabetic neuropathy and data from human and animal studies suggest that glucose-derived oxidative stress plays an important role in impaired neurotrophic support (Greene et al. 1999). Both chronic and acute hyperglycemia cause oxidative stress in the peripheral nervous system that can promote the development of diabetic neuropathy (Vincent et al. 2004). Similarly, studies on hepatitis C virus-associated mixed cryoglobulinemia vasculitis neuropathy (Saadoun et al. 2007), alcohol-related neuropathy (Kaur et al. 2017) oxaliplatin-induced sensory neuropathy (Joseph et al. 2008), docetaxel-induced neuropathy (Mir et al. 2009), and placlitaxel-induced neuropathy (Duggett et al. 2016) show that increased oxidative stress plays a key role and in fact might also explain the increased prevalence of pain in such neuropathies (Brozou et al. 2018; Artemiadis and Zis 2018). To our knowledge, ours is the first study to investigate the role of oxidative stress in CIAP.

In our study, we chose to analyze 8H and PC in patients’ sera.

Protein carbonyls are among the most used markers for protein oxidation and more in general for oxidative stress (Dalle-Donne et al. 2003). In addition they are extremely stable in plasma in the proper storing conditions (Stadtman and Levine 2003).

In a recent study, Almogbel and Rasheed showed that patients with diabetic neuropathy show higher levels of protein carbonyl compared to controls (Almogbel and Rasheed 2017).

Similarly, 8-hydroxydeoxyguanosine is regarded as a marker for reactive oxidative species (ROS)–induced DNA-modification which can be found in both serum and urine (Lunec et al. 2002). Also, it has been shown that nerves in rat subjects with diabetes contain an increased number of macrophages and 8-hydroxydeoxyguanosine-positive cells in the endoneurium (Mizukami et al. 2011). Interestingly, the latter are significantly suppressed by methylcobalamin treatment (Mizukami et al. 2011).

The fact that oxidative stress is increased in patients with neuropathy and might play an even more important role in those patients where no cause for their neuropathy is identified (CIAP) broadens future research fields. Interventional studies of agents reducing oxidative stress are needed to show whether such interventions lower the progression rate of neuropathy. Already such interventions have been tried in both animal models with molecules such as carvedilol (Areti et al. 2017) and resveratrol (Kumar et al. 2007) and human studies with compounds such as calmangafodipir (Glimelius et al. 2018).

Our results should be interpreted with some caution, given the limitations of our design. This is a cross-sectional study based on patients attending a specialized clinic, and the results may not be generalizable to other settings. Our sample size was small. Although this study was controlled, a larger future study should either include a healthy control group or a group of patients with genetic neuropathy to confirm our findings. A comparison with diabetic neuropathy patients, a group where oxidative stress also plays a role, will also be interesting. Furthermore, our cohort included patients with large fiber axonal peripheral neuropathies only. Pure small fiber neuropathy is another area that merits further consideration.

In conclusion, oxidative stress is increased in patients with CIAP. Oxidative stress markers such as PC or 8H have a potential as biomarkers in such patients. Lowering oxidative stress might be a treatment option for patients with neuropathy and, therefore, prospective interventional multicenter longitudinal studies are needed.
